# Multiuser wireless network enhancement via an innovative rime optimization search strategy

**DOI:** 10.1371/journal.pone.0323138

**Published:** 2025-06-02

**Authors:** Wafaa Alsaggaf, Mona Gafar, Shahenda Sarhan, Abdullah M. Shaheen, Ahmed S. Alwakeel

**Affiliations:** 1 Department of Information Technology, Faculty of Computing and Information Technology, King Abdulaziz University, Jeddah 21589, Saudi Arabia; 2 Department of Computer Engineering and Information, College of Engineering, Wadi Ad Dwaser, Prince Sattam Bin Abdulaziz University, Al-Kharj, Saudi Arabia; 3 Machine Learning and Information Retrieval Department, Artificial Intelligence, Kafrelsheikh University, Kafrelsheikh, Egypt; 4 Computer Science Department, Faculty of Computers and Information, Mansoura University, Mansoura, Egypt; 5 University of Economics and Human Sciences, Warsaw, Poland; 6 Department of Electrical Engineering, Faculty of Engineering, Suez University, Suez, Egypt; Beijing Institute of Technology, CHINA

## Abstract

This paper introduces an Improved Rime Optimization Algorithm (IROA) designed to maximize achievable rates in multiuser wireless communication networks equipped with Reconfigurable intelligent surfaces (RISs). The proposed technique incorporates the Quadratic Interpolation Method (QIM) into the classic Rime Optimization Algorithm (ROA), which improves solution diversity, facilitates broader exploration of the search space, and enhances robustness against local optima. Finding the ideal quantity and positioning of RIS components to optimize system performance is the main goal of the optimization framework. Two objective models are taken into consideration: one that maximizes the lowest achievable rate in order to prioritize fairness, and another that maximizes the average achievable rate for all users. The performance of IROA is evaluated on systems with 20 and 50 users and compared against established algorithms such as Differential Evolution (DE), Particle Swarm Optimization (PSO), Grey Wolf Optimizer (GWO), Augmented Jellyfish Search Optimization Algorithm (AJFSOA), and Jellyfish Search Optimization Algorithm (JFSOA). Results demonstrate that the proposed IROA achieves relative performance improvements ranging from 5% to 46% across different scenarios and objective models. In the 20-user case with the first objective model, IROA achieves improvements of 28.02%, 42.07%, 46.54%, 1.74%, 35.46%, and 25.95% compared to AJFSOA, JFSOA, PSO, ROA, GWO, and DE, respectively, in terms of average achievable rate. Similarly, for the second objective model, IROA achieves relative improvements of 5.94%, 13.29%, 14.55%, 7.1%, 15.97%, and 46.26% over ROA, DE, PSO, AJFSOA, JFSOA, and GWO, respectively, in terms of minimum achievable rate. On contrary, the IROA shows lower standard deviation compared to the current ROA. However, the proposed IROA achieves superior performance over ROA in terms of the best, mean and worst objective outcomes. These findings demonstrate that in RIS-assisted wireless communication networks, the suggested IROA achieves strong flexibility and reliable performance benefits across a range of multiuser optimization tasks.

## Introduction

In the era of Beyond 5th-generation cellular network (B5G) and developing sixth-generation cellular networks (6G) communication systems, delivering larger capacity, enhanced dependability, and energy efficiency in wireless networks has become critical [1–3]. Reconfigurable intelligent surfaces (RISs) are a disruptive technology that allows for dynamic customization of the wireless environment to improve signal quality and system performance [4]. RISs are made up of programmable metasurfaces that may reflect, refract, or absorb electromagnetic waves, giving you exact control over signal transmission. This capacity places RISs as a cornerstone technology for constructing Smart Radio Environments (SREs) that adapt to varied communication scenarios [5–8].

Despite RISs’ potential, their practical deployment faces significant challenges, including proper placement, configuration, and the construction of efficient algorithms for their operation [9–11]. The performance of RIS-assisted wireless networks is strongly dependent on optimizing parameters like RIS placements and phase shifts. Traditional optimization approaches, such as Differential Evolution (DE) and Particle Swarm Optimization (PSO), struggle to strike a balance between exploration and exploitation in RIS optimization’s high-dimensional search space.

### Related Work

In order to properly capitalize on the benefits resulting from the implementation of RISs, it is important to possess a thorough comprehension of all facets of RIS- aided wireless communication systems. Of them, the most important one would be designing RIS phase changes with the goal of manipulating incoming radio waves in a way that maximizes the benefits mentioned above. Thus, creating algorithms that maximize achievable rates is especially important when it comes to RIS-assisted communications. The goal of the majority of this field’s research has been to maximize achievable rates for point-to-point multiple-input multiple-output (MIMO) communications. Although publications like those found in [12, 13] provide effective techniques for transmit covariance matrix optimization, they mostly focus on single-user MIMO situations. On the other hand, [14] examined how to optimize the sum rate while employing millimeter-wave (mmWave). The optimization approaches shown in [14] are restricted to single-user contexts, yet they produce near-optimal achievable rates with little computational and hardware complexity.

In [15], researchers addressing discrete signaling showed that the cutoff rate may be used as a more manageable optimization parameter to successfully optimize achievable rates in RIS-aided systems. Moreover, the research in [16] explored how a modest number of active components added to the RIS may improve spectral efficiency. They maximized the possible rate by working together to optimize the RIS matrix and the precoding vectors. To approach the nonconvex problem, many strategies are utilized in [17]: maximum ratio transmission, ideal power allocation, and Currant penalty. In Internet of Things (IoT) networks, this study aims to maximize the achievable rate in RIS-assisted uplink MIMO. RIS phase shifts, power distribution, and base station beamforming design are all collaboratively optimized to improve spectral efficiency with many mobile IoT devices now in use [18].

Using received signal strength indicator (RSSI) measurements for a cellular user in a millimeter-wave network, the authors of [19] offer RISs to examine the efficacy of range estimation. Firstly, they delineate the best practice for RIS deployment, which lessens the likelihood of totally preventing the user from accessing both the RIS connection and the BS. Next, based on certain constraints, the authors described a range estimation method. In [20], authors discussed localization algorithms based on near-field (NF) technology and suggested RIS as a specific method for resolving issues. Accurately and simultaneously localizing many energy-constrained devices is a necessary condition for many location-based Internet of Things applications. The ergodic achievable rate’s approximate values are first calculated using Jensen’s inequality and majorization theory. After that, an alternating optimization-based method is proposed to optimize the ergodic achievable rate by concurrently building the transmit covariance matrix at the base station and the reflection coefficients at the RIS [21]. A achievable sum-rate maximization problem arises from the optimization of the RIS phase shift, resource distribution, and UAV placement together. Using the block coordinate descent (BCD) method [22], the original problem is divided into three smaller problems and solved to avoid the non-convexity caused by the coupling of variables.

The authors in [23] provided an intelligent metaheuristic version of an improved artificial ecosystem optimizer (EAEO) for a beamforming optimization framework in an integrated sensing and communication (ISAC) system that has been integrated with RIS [24, 25]. The ISAC system is designed to maximize signal transmission over the RIS and improve overall performance by optimizing the SNR. To improve the quality of the solutions in multidimensional and nonlinear optimization situations, the suggested EAEO version integrates a Fitness-Distance-Balance Model (FDBM) with the basic Artificial Ecosystem Optimizer (AEO).

In [26], authors investigated the complementarity of digital twins (DTs) to successfully deploy the metaverse in 6G networks. To be more specific, they study how a DT-assisted RIS-based network architecture can significantly enhance network latency and reliability for 6G metaverse implementation. In [27], an energy-efficient downlink communication system incorporating multiple aerial RISs (ARISs) is investigated. The addition of many ARISs enables UEs and BS to interact more easily. The joint optimization issue of the multiple ARIS-assisted communication system’s power regulation, phase shift, and ARIS reflecting element on/off states is then established.

The authors of [28] described a blockchain-based architecture for transmitting and storing information, enabling for safe knowledge management in the intelligent IoT. Their first permissioned blockchain-based decentralized and trustworthy knowledge storage system incorporates on-chain encrypted knowledge storage and an improved Delegated Proof of Stake (DPoS) consensus mechanism. In [29], a new wirelessly powered edge intelligence (WPEG) architecture was presented. The goal was to create robust, reliable, and long-lasting edge intelligence using energy harvesting (EH) techniques. To protect peer-to-peer (P2P) energy and knowledge exchange in our system, they first created a permissioned edge blockchain. The authors of [30] created a multiple access approach for next generation multiple access (NGMA) in wireless communication systems using various RISs. They initially looked into the relationship between the efficiency and complexity of the RIS phase setup and the design of NGMA schemes, keeping in mind the real-world scenario of stationary users interacting with mobile users. They then created a media access control (MAC) protocol that incorporates RISs and presented a multiple access framework for RIS-assisted communication systems.

The authors of [31] devised a generic formulation of the ergodic capacity for RIS-aided communication systems, taking into account the radiative properties of RIS. This equation accounts for both the LOS and NLOS connections. This introduces a new level of freedom in optimizing RIS-aided wireless channels. Using the channel model, we investigate the RIS deployment strategy, which includes RIS rotation and placement optimization. The authors of [32] suggested a conjugate gradient and particle swarm optimization (CG-PSO) approach to optimize both RIS phase shifts and ABS heights. The conjugate gradient (CG) under fixed ABS altitude and changing transmit power is used to calculate an appropriate RIS phase shift. Finally, they used PSO to determine the best ABS altitude, resulting in a greater sum rate at the optimal RIS phase shift.

The Rime Optimization Algorithm (ROA) is a nature-inspired metaheuristic which was recently introduced by H. Su *et al*. [33]. It simulates the evolutionary behavior of rime particles, transitioning between soft and hard forms to adapt to environmental conditions. ROA operates through two core processes: the Hard-Rime Puncture Process (HRPP) and the Soft-Rime Search Process (SRSP), which emulate distinct strategies for exploring and exploiting the search space. In this study, we propose an improved version of ROA by integrating the Quadratic Interpolation Method (QIM). Unlike the traditional ROA, which relies solely on the best-performing agent, the proposed IROA incorporates data from three different rime agents. This approach introduces greater solution diversity and enhances the exploration of the search space by leveraging randomization and permutations, leading to a more robust optimization process. The proposed IROA technique is specifically designed to determine the maximum achievable rate in multiuser wireless communication networks equipped with Reconfigurable Intelligent Surface (RIS).

### Motivation

The fundamental impetus for this research stems from the limits of current optimization techniques in dealing with the particular issues of multiuser RIS-assisted wireless networks. RISs have developed as a transformative technology, significantly improving wireless communication performance. Optimizing RIS parameters, such as location and phase shifts, is a difficult effort because to the problem’s large dimensionality and non-convexity. Traditional population-based optimization techniques, such as DE, PSO, and others, have shown various shortcomings: (1) Limited Exploration Capabilities: These algorithms frequently fail to search the solution space completely, resulting in poor solutions and a higher probability of being locked in local optima; (2) Slow Convergence: Many present approaches require numerous iterations to converge, making them computationally expensive for large-scale and real-time applications. These limitations highlight the need for a more advanced, adaptive optimization algorithm. The suggested Improved Rime Optimization Algorithm (IROA) overcomes these issues by including the QIM.

### Main contribution

This research addresses the issues of maximizing the achievable rate in RIS-assisted networks while maintaining user fairness, a significant concern in earlier optimization models. For this purpose, an innovative IROA has been designed to improve the performance of multiuser wireless communication systems with RISs. This innovation increases exploration diversity and exploitation efficiency, allowing the algorithm to locate globally optimal solutions with greater confidence and speed. Furthermore, IROA introduces a fairness-driven goal function that maximizes the lowest achievable rate, assuring equal performance for all users. This is especially important in cases involving dense user populations or varied network topologies, where traditional algorithms fail to strike a compromise between fairness and system capacity.

By addressing these major deficiencies, the proposed IROA seeks to advance the state-of-the-art in RIS optimization, providing a scalable and robust solution for next-generation wireless networks. This study provided practical insights on efficiently utilizing RIS technology, particularly in situations where fairness and efficiency are critical.

The key aspects of the contributions are as follows:

1. Enhanced Optimization Framework: We propose enhancing the classic Rime optimization Algorithm by integrating the QIM. This innovation adds more diversity to the search process and allows for a more robust exploration of the solution space, overcoming the limits of standard optimization strategies, which frequently become locked in local optima.
2. Two Objective Models: We present two alternative objective models for maximizing the achievable rate in multiuser RIS-assisted systems. The first model seeks to maximize the average achievable rate across all users, resulting in optimal overall system performance. The second model focuses on maximizing the lowest achievable rate for each user, assuring fairness and prioritizing the user with the lowest performance.
3. Effectiveness and validation of IROA: Extensive simulations show that the proposed IROA outperforms existing algorithms, such as the traditional ROA [33], DE [34, 35], PSO [36, 37], Grey Wolf Optimizer (GWO) [38, 39], Augmented Jellyfish Search Optimization Algorithm (AJFSOA) [40], and Jellyfish Search Optimization Algorithm Jellyfish Search Optimization Algorithm (JFSOA) [41], in terms of both average and minimum rates. The results demonstrate IROA’s superior ability to balance system performance and fairness in RIS-assisted wireless networks.

To ensure a thorough performance evaluation, the proposed IROA is compared against a number of well-known metaheuristic optimization algorithms that have recently been applied to RIS-assisted wireless communication challenges. PSO [42, 43] and DE [44, 45] are commonly employed for optimizing RIS phase shifts and placement due to their efficient search processes and capability to tackle high-dimensional issues. Furthermore, nature-inspired algorithms, such as the AJFSOA [40] and JFSOA [46], have shown promising results in improving spectral efficiency, signal coverage, and energy efficiency in RIS-assisted multiuser networks. These algorithms reflect cutting-edge techniques to RIS optimization, making them appropriate benchmarks for assessing IROA’s performance. The results of this study show that IROA consistently outperforms these approaches in terms of feasible rate maximization, fairness, and convergence speed, confirming its robustness in RIS-assisted wireless communication networks.

The rest of the paper is organized as follows. The System model section provides an explanation of the system model. The recommended IROA method for maximizing the achievable rate is given in the Proposed IROA incorporating QIM for performance improvement in wireless communication systems section. The Numerical results section offers numerical results to verify the performance of the recommended algorithm. The final section concludes the article.

*Notation:* We represent matrices as X and column vectors as x in lowercase and uppercase, respectively. The equivalent pseudo-inverse, transpose, conjugate transpose (Hermitian), and inverse for each X are denoted, respectively, by the symbols $X^{†}$, $X^{T}$, $X^{H}$, and $X^{-1}$. tr(X) is the trace function of a matrix X. ‖.‖ is the representation of the Euclidean norm. The notation for a circularly symmetric complex Gaussian random vector is x~𝒞𝒩(μ,φ), where μ represents the mean and φ the covariance matrix. ℂ is the symbol for the whole set of complex numbers. ${\mathbb{C}}^{N\times 1}$ and ${\mathbb{C}}^{N\times M}$ denote the generalizations for matrices and vectors, respectively, in this notation. $I_{M}$ is the identity matrix of size M×M.

## System model

**Fig 1:**
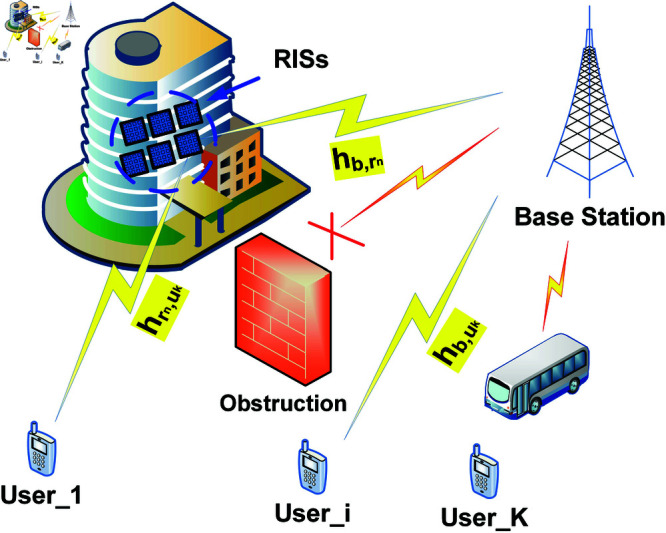
System model.

As seen in Fig 1, we propose a multi-RIS-aided wireless communication system for outdoor situations. In this system, a single-antenna base station (BS) and *K* single-antenna user equipment (UE) are connected by *N* equal-size RISs in orthogonal time slots. It is assumed that every RIS that is installed on the ceiling is a homogeneous planar array with *M* reflecting components.

### Communication channel model

As often considered in the literature, for example, [47–52], there is accurate channel state information (CSI) for the links between the BS, users, and RIS elements. This reflects that the base station has full knowledge of the channel conditions between all users and the RIS, allowing for optimal resource allocation and interference mitigation. Under this consideration, the interference term from other users is negligible and can be effectively managed through appropriate power control and RIS configuration. $\phi_{n}=\{e^{j\theta_{n,1}},e^{j\theta_{n,2}},...,e^{j\theta_{n,M}}\}$ represents the phase shift coefficient vector of RIS *n*, where $\theta_{n,r}$ represents the phase shift of element *r* in RIS *n*. Furthermore, we assign the channel vectors in this manner: For the channel vector from UE *k* to RIS *n*, $h_{b,r_{n}}\in {\mathbb{C}}^{M\times 1}$; for the channel vector from RIS *n* to the BS, $h_{r_{n},u_{k}}\in {\mathbb{C}}^{1\times 1}$; and for the channel vector from UE *k* to the BS, $h_{b,u_{k}}\in {\mathbb{C}}^{1\times 1}$. The RIS can help construct channels that are dominated by LoS propagation with a few scatters since it can be put on the walls of tall structures. For the UE-RIS and RIS-BS channels, we thus employ the Rician fading model presented in [53–55]. The components of the LoS can be expressed using an array response vector. The array response vector for the UE-RIS connection may be described using the azimuth and elevation angles of arrival (AoA) at the RIS from the UE. The array response vector for the RIS-BS link may be defined by the azimuth and elevation angles of departure (AoD) from the RIS towards the BS. The non-line-of-sight (NLoS) components belong to the distribution of 𝒞𝒩(0,1) [56–58] and are composed of complex Gaussian random variables that are independently and identically distributed (i.i.d). Consequently, $h_{b,r_{n}}$ is written as [53]

$$h_{b,r_{n}}=P_{b,r_{n}}\left(\sqrt{\frac{\epsilon }{\epsilon +1}}r_{n}(\gamma_{n},\xi_{n})+\sqrt{\frac{1}{\epsilon +1}}d_{b,r_{n}}\right)$$ (1)

$P_{b,r_{n}}$ indicates the path-loss between RIS *n* and the BS, and ϵ is the Rician factor that accounts for the ratio of the LoS power to the NLoS power. The array response of RIS *n* is represented by the symbol $r_{n}\in {\mathbb{C}}^{M\times 1}$; the azimuth angle is $\gamma_{n}$, and the elevation angle of departure is $\xi_{n}$ for the link between RIS *n* and the BS. The direct channel elements, $d_{b,r_{n}}$, are chosen from CN(0,1). Likewise, $h_{r_{n},u_{k}}$ is written as [53]

$$h_{r_{n},u_{k}}=P_{r_{n},u_{k}}\left(\sqrt{\frac{\epsilon }{\epsilon +1}}r_{n}(\gamma_{n}^{\prime },\xi_{n}^{\prime })+\sqrt{\frac{1}{\epsilon +1}}d_{r_{n},u_{k}}\right)$$ (2)

where the symbols $\xi_{n}^{\prime }$ and $\gamma_{n}^{\prime }$ represent the elevation angle and azimuth angle for the connection between RIS *n* and UE *k*, respectively. The following is the direct channel between user *k* and the BS

$$h_{b,u_{k}}=P_{b,u_{k}}d_{b,u_{k}}$$ (3)

The path-loss between UE k and the BS is shown in this instance by $P_{b,u_{k}}$. The signal received at the BS form RIS *n* is provided by

$$y_{BS,n}=({𝐡}_{b,r_{n}}^{H}\Phi_{n}h_{r_{n},u_{k}}+h_{b,u_{k}})x+n$$ (4)

The transmitted symbol *x* has a power of *p*_*k*_, and $\Phi_{n}=diag(\phi_{n})$. The effective channel is ${𝐡}_{b,r_{n}}^{H}\Phi_{n}h_{r_{n},u_{k}}$, which includes RIS phase shift. The noise is represented by *n* with $NC(0,\sigma^{2}$), and the straight path is $h_{b,u_{k}}$. For user *k*, the received SNR ^1^ is provided by

$$SNR_{k}=\left(\frac{p_{k}|\alpha_{b,u_{k}}h_{b,u_{k}}+\sum_{n=1}^{N}\alpha_{b,r_{n}}h_{b,r_{n}}^{H}\Phi_{n}h_{r_{n},u_{k}}{|}^{2}}{\sigma^{2}}\right)$$ (5)

The achievable sum rate of user *k* as displayed

$$R_{k}=𝐄\{\log_{2}(1+SNR_{k})\}$$ (6)

Where the parameters $\alpha_{a,n}$ and $\alpha_{a,k}$ satisfy

$$\alpha_{b,r_{n}}=\left\{ \begin{array}{cc} 0 & \text{if the link between RIS n}andBSisblocked\\ 1 & \text{otherwise} \end{array}\right.$$ (7a)

$$\alpha_{b,u_{k}}=\left\{ \begin{array}{cc} 0 & \text{if the link between RIS n}andBSisblocked\\ 1 & \text{otherwise} \end{array}\right.$$ (7b)

### Problem formulation

In this study, we provide a technique for optimizing the amount, distribution, and RIS reflection patterns in a wireless communication system with RIS assistance simultaneously. To show how effective the suggested strategy is, the final parameters are assessed. The proposed methodology uses two different objective models to improve the performance of multiuser communication systems.

#### Proposed objective functions.

To achieve a balanced performance among several participants, the first step is to maximize the average achievable rate of each participant. This method, which may be expressed as follows, prevents excessive values in any user

$$\underset{N,\{y_{n},z_{n}\},\xi_{n}}{maximize}\;\;\;Ob_{1}=\frac{\sum_{k=1}^{K}R_{k}}{K}$$ (8)

An alternative approach is to emphasize improving those in the worst condition to the fullest extent possible by maximizing the lowest rate that each user can achieve.

$$\underset{N,\{y_{n},z_{n}\},\xi_{n}}{maximize}\;\;\;Ob_{2}=min\{R_{k}\},k=1:K$$ (9)

#### Proposed constrains.

The following practical constraints should be upheld in order to maximize the previously indicated objective models:

$$x_{min}\leq x_{n}\leq x_{max},\forall n\in \{1:N\}$$ (10a)

$$z_{min}\leq z_{n}\leq z_{max},\forall n\in \{1:N\}$$ (10b)

$$min\{|x_{n}-x_{c}|,|z_{n}-z_{c}|\}\geq L,\forall n,c\in \{1:N\}$$ (10c)

$$N_{min}\leq N\leq N_{max}$$ (10d)

where *L* is the size length of each RIS and $\left\{x_{n},z_{n}\right\}$ is the position of RIS *n*. The location limitations of the RISs are demonstrated by the constraints (11) and (12). To make sure that RISs do not overlap, we use (13); similarly, (14) guarantees that the number of RISs falls between [$N_{min},N_{max}$].

## Proposed IROA incorporating QIM for performance improvement in wireless communication systems

In this section, the standard ROA and the proposed IROA are highlighted and their main mathematical formulation are displayed. Also, the proposed algorithm’s design and its operational differences from ROA are explicitly described in addressing the optimization challenges in RIS-assisted wireless networks.

### Standard ROA

ROA is rooted in natural phenomena, specifically aimed at imitating the growth of rime particles under various environmental conditions. It mimics the relevant environmental factors through the implementation of the SRSP and the HRPP [33]. The HRPP represents the destructive aspects observed during rime formation, where the effectiveness of particle movement affects the fitness function. On the other hand, the SRSP models the rime formation in a windy climate [59, 60]. Moreover, a crossover operator is utilized to facilitate information exchange among the particles, contributing to improved convergence.

The initialization of the rime population involves a random searching procedure [61]. During this initial phase, the position of the rime (*P*_*mn*_) as follows:

$$P_{mn}(tr=0)=PL_{m}+z_{1}\times (PU_{m}-PL_{m});\;\;\;n=1:Dim,\;\;m=1:P_{s}$$ (11)

where *P*_*s*_ denotes the number of particles in the population, the problem’s dimension is indicated by *Dim*, *PL*_*m*_ and *PU*_*m*_ refer to the dimensional boundaries, and *z*_1_ represents a randomly selected value from the interval (0,1). Then, the SRSP is activated, wherein rime particles traverse the searching area. Therefore, in the next iteration (tr+1), the position of every rime particle is updated through the SRSP, defined as $P^{*}(tr\;+\;1)$, as follow:

$$P^{*}(tr+1)=P\_best_{n}(tr)+PA_{n}(tr),\;\;\;if\;\;\;z_{2}<\sqrt{\frac{tr}{tr_{max}}}$$ (12)

In the given context, the notation $P\_best_{n}(tr)$ represents the dimension (*n*) corresponding to the best solution found so far (P_best) within the current iteration (*tr*), with *tr*_*max*_ indicating its maximum value. The symbol *z*_2_ represents a randomly selected value from the interval [0,1]. *PA*_*n*_(*tr*) is an artificial rime particle that can be created [62] using the following expression:

$$PA_{n}(tr)=z_{3}\times cos(\theta (tr))\times [H\times (PU_{n}-PL_{n})+PL_{n}]\times \beta (tr)$$ (13)

where *z*_3_ represents another randomly number from the range [-1,1]. *H* denotes the adhesion degree, which is also determined by a randomly number from the interval [0,1]. The variables β(tr) and θ(tr) mimic external conditions that represent environmental factors, and their models are defined as follows:

$$\theta (tr)=\frac{\pi }{10}\times (\frac{tr}{tr_{max}})$$ (14)

$$\beta (tr)=1-\left(\frac{1}{\Psi }\times (round(\frac{\Psi \times tr}{tr_{max}}))\right)$$ (15)

Accordingly, as stated in [33], the factor Ψ determines the division of the step function and defaults to a value of five.

In contrast, under high-wind conditions, the HRPP is repeated to simulate the simpler and more orderly growth of hard-rime particles. Therefore, the position of each rime particle can be updated and denoted as $P\_New_{mn}(tr+1)$, using the following expression:

$$P\_New_{mn}(tr+1)=\left\{\begin{array}{cc} P\_best_{n}(tr), & \text{if}z_{4}<F^{NoM}(P^{*}(tr)),\\ P_{mn}^{*}(tr+1), & \text{Else} \end{array}\right\},\;\;\;\;n=1:Dim,\;\;m=1:P_{s}$$ (16)

where *z*_4_ represents a randomly selected number from the interval [0, 1]. $\text{Fit}({\text{P}}^{*}(\text{tr}))$ is the fitness value of $\left({\text{P}}^{*}(\text{tr})\right)$ while ${\text{F}}^{\text{NoM}}({\text{P}}^{*}(\text{tr}))$ represents its related normalized value, defined as follows:

$$F^{NoM}(P^{*}(tr))=\frac{Fit(P^{*}(tr))}{\sqrt{\sum_{m=1}^{P_{s}}(F(P^{*}(tr)){)}^{2}}};\;\;\;m=1:P_{s}$$ (17)

The ROA procedure involves generating a new particle solution, evaluating its fitness level, and comparing it with the previous solution. If the new obtained fitness is better, it replaces the inferior solution. Throughout iterations, this process continuously replaces particles to ensure the beneficial evolution of the population. The pseudo code for the standard ROA is depicted in Algorithm 1 which introduces a systematic approach to initialize and evolve a population of particles to find the optimal solution for a given problem. The algorithm begins by initializing several parameters. The population of particles is then initialized using a random search procedure defined by Equation 11, ensuring that each particle’s position is randomly set within the defined boundaries. Initially, the fitness of each particle is evaluated using Equation 17, which normalizes the fitness values of the particles. This helps in comparing the relative quality of different particles’ positions within the search space. The main optimization process occurs within a loop. For each particle, the SRSP and HRPP are applied in the standard ROA. After updating the positions, the fitness of each particle is re-evaluated. A greedy search technique is employed to compare the fitness of the new position with the previous one. If the new position’s fitness is better, it replaces the old position, ensuring continuous improvement in the population’s overall quality. Finally, after completing all iterations, the algorithm returns the best solution found. This solution represents the particle with the highest fitness value achieved during the optimization process.

**Algorithm 1. Pseudo Code for ROA**

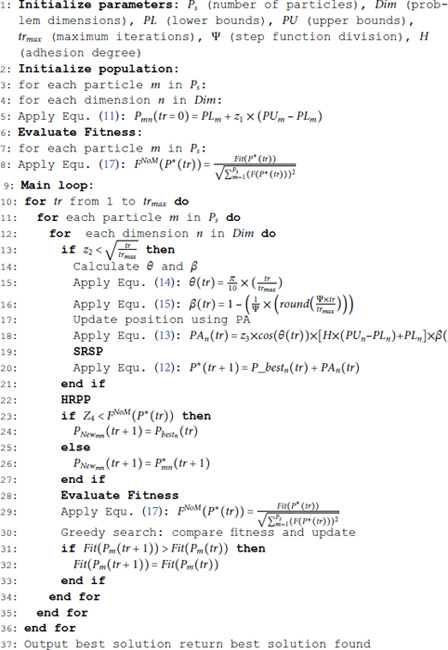



## Proposed IROA

To enhance population diversity and improve the exploration capabilities of the ROA, this research proposes the integration of an IROA approach with the QIM, which is a local search technique designed to fit a parabolic function to a given curve and identify its extremities. By incorporating QIM into the ROA, higher diversity is introduced, and non-linear modifications are enabled through quadratic operations. The QIM approach refines the solution particle (*P*_*m*_(*tr*)) around itself and two randomly selected neighbors (*P*_*R*1_(*tr*) and *P*_*R*2_(*tr*)).

Consequently, the position of each rime particle can be updated using the QIM strategy, represented as $P\_New_{m}(tr\;+\;1)$, according to the following expression:

$$P\_New_{m}(tr+1)=\frac{A(tr)+B(tr)+C(tr)}{2\times (a(tr)+b(tr)+c(tr))};\;\;\;m=1:P_{s}$$ (18)

where

$$A(tr)=(P_{R1}(tr{)}^{2}-P_{R2}(tr{)}^{2})\times Fit(P_{m}(tr))$$ (19)

$$B(tr)=(P_{R2}(tr{)}^{2}-P_{m}(tr{)}^{2})\times Fit(P_{R1}(tr))$$ (20)

$$C(tr)=(P_{m}(tr{)}^{2}-P_{R1}(tr{)}^{2})\times Fit(P_{R2}(tr))$$ (21)

$$a(tr)=(P_{R1}(tr)-P_{R2}(tr))\times Fit(P_{m}(tr))$$ (22)

$$b(tr)=(P_{R2}(tr)-P_{m}(tr))\times Fit(P_{R1}(tr))$$ (23)

$$c(tr)=(P_{m}(tr)-P_{R1}(tr))\times Fit(P_{R2}(tr))$$ (24)

where *Fit*(*P*_*m*_(*tr*)), *Fit*(*P*_*R*1_(*tr*)), and *Fit*(*P*_*R*2_(*tr*)), denote the fitness scores of the present particle (*m*) and the two randomly selected neighbors (*R*1 and *R*2), respectively.

**Fig 2:**
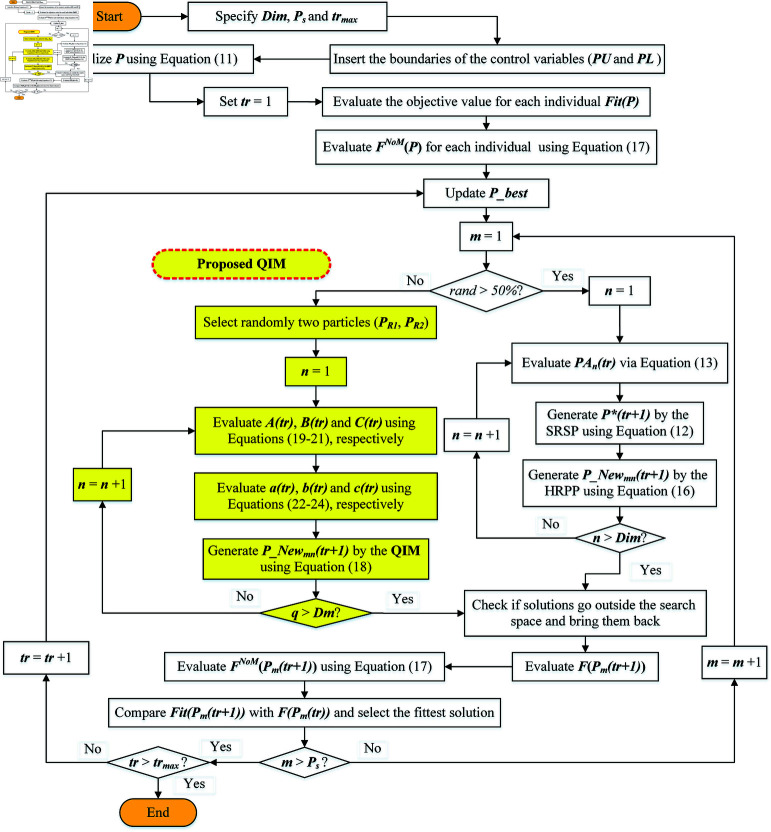
IROA flow chart.

The method includes employing a positive greedy search technique to compare fitness ratings prior to and following upgrades. The HRPP, SRSP, or QIM are utilized to generate new positions for rime particles. If the updated fitness value is superior, the suboptimal solution is replaced by the optimal one, thereby improving the overall quality of the global solution. This method actively replaces agents throughout upgrades to guarantee optimal population evolution. Fig 2 shows the flow chart of the proposed IROA approach. Also, the pseudo code for the presented IROA incorporating the QIM is depicted in algorithm 2. In the proposed IROA, for each particle, a random decision (if “*rand*>0.5”) determines whether to follow the SRSP and HRPP or to use the QIM-enhanced IROA. The application of the QIM-enhanced IROA is activated to enhance the particle positions. For each particle, two random neighbors (*P*_*R*1_(*tr*) and *P*_*R*2_(*tr*)) are selected from the population. The values *A*(*tr*), *B*(*tr*), *C*(*tr*) are calculated using Equations 18, (23) and (24), respectively, based on the fitness of the current particle and its neighbors. Similarly, the values *a*(*tr*), *b*(*tr*), and *c*(*tr*) are calculated using Equations 21, (26) and (27). These intermediate calculations are used to update the particle’s position using Equations 18Equation 18, which ensures that the new position is derived from a parabolic interpolation of the fitness landscape. After updating the positions, the fitness of each particle is re-evaluated. A greedy search technique is employed to compare the fitness of the new position with the previous one. If the new position’s fitness is better, it replaces the old position, ensuring continuous improvement in the population’s overall quality. Finally, after completing all iterations, the algorithm returns the best solution found. This solution represents the particle with the highest fitness value achieved during the optimization process.

Compared to traditional optimization algorithms like DE, PSO, and ROA, IROA offers several advantages. First, QIM prevents premature convergence by maintaining a diverse set of candidate solutions throughout the optimization process. Second, IROA achieves faster convergence rates due to its adaptive mechanisms. Finally, the algorithm is scalable to high-dimensional optimization problems often encountered in RIS-assisted networks.

**Algorithm 2. Pseudo Code for IROA**

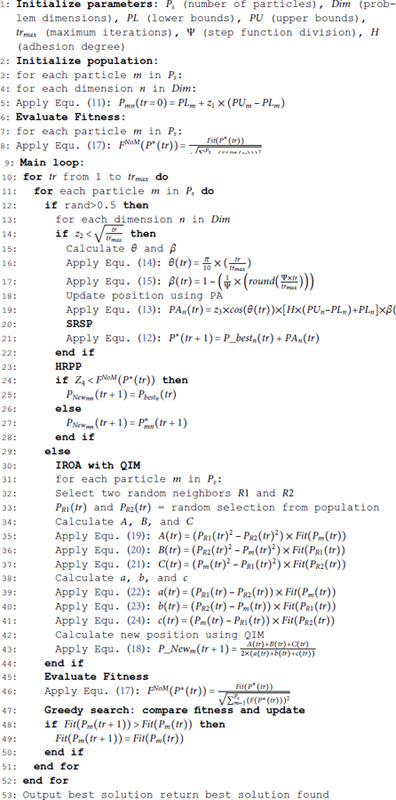



## Numerical results

Here, we conduct many simulations to evaluate the superiority of the IROA algorithm over the other approaches. The goal of all these approaches is to optimally combine the number and placement of RISs. The following subsection shows the experimental setting.

### Experimental settings

In this subsection, we present a full description of the setup that was utilized to assess the performance of the proposed IROA algorithm in RIS-assisted multiuser wireless communication networks.

#### Simulation environment.

The simulations are conducted in a 3D environment, with the communication system consisting of a BS and *K* users each equipped with a single antenna. The base station and users are situated outside, with various distances and angles between them. The RISs are placed in the system to assist with signal propagation. Each RIS is formed from 100 reflecting components, which are positioned optimally during the experiments. The BS and RISs are considered to be static, whereas the users are assigned at random within a particular area. The users are dispersed randomly within a square with vertices of [0, 0, 0], [0, 10, 0], [10, 10, 0], and [10, 10, 0] m. The RISs are arranged within the square with vertices of [0, 0, 10], [0, 10, 10], [10, 0, 10], and [10, 10, 10] m. Moreover, the BS’s coordinates were set to [5, -10, 1] m. Four vertices, located at [0, 0, 0], [0, 0, 2.25], [10, 0, 2.25], and [10, 0, 0] m, were used to determine the wall’s placement (c.f. Fig 1).

All simulations were run using MATLAB (R2023a) on a computer equipped with an Intel Core i7-470K CPU processor, 16 GB of RAM, and Windows 11 operating system. The proposed approach was implemented and numerically evaluated using MATLAB’s built-in optimization toolbox and custom scripts. The simulation parameters and settings were carefully chosen to produce accurate and consistent results.

#### System parameters.

Table 1 presents the values of the system parameters used in simulation. To ensure the study’s reproducibility and practical applicability, we carefully selected essential simulation parameters from generally accepted models and real-world deployment situations in RIS-assisted wireless networks. The Rician Factor was set to 10, which represents typical urban and suburban situations in which both Line-of-Sight (LOS) and non-line-of-sight (NLOS) components impact signal propagation. This option is consistent with earlier research into RIS-based communication systems. The Noise Power was computed using an SNR level of –23 dB, which is consistent with conventional assumptions in wireless network optimization studies. Furthermore, the Path Loss Exponent was increased to 2.2 for UE-RIS-BS links and 4 for direct UE-AP lines to simulate realistic signal attenuation in crowded communication settings. These values have been referenced from recent literature to ensure consistency with existing work and enhance the reproducibility of our results.

**Table 1:** System parameters

Parameter	Value
Number of Users (K)	20 and 50 users
RIS Elements (M)	100 reflective elements per RIS
Rician Factor (ϵ)	10
Transmit Power (*p*)	1 mW
Noise Power ($\sigma^{2}$)	Corresponding to SNR = -23 dB
Path Loss Exponent (β)	2.2 (UE-RIS-BS), 4 (UE-AP)
Number of RISs	1 to 10
Placement Constraints	No overlapping, within boundaries

#### Optimization problem.

The optimization focuses on maximizing the achievable rates under two different objective models: (1) Objective Model 1: Maximizing the average achievable rate of all users. (2) Objective Model 2: Maximizing the minimum achievable rate, ensuring fairness across the network by improving the rate for the user with the lowest achievable rate.

#### Algorithms compared.

To evaluate the performance of the proposed IROA, we compare it against several well-known optimization algorithms, including: DE, PSO, Grey Wolf Algorithm (GWA), Traditional ROA, AJFSOA, and JFSOA.

#### Performance metrics.

The performance of each algorithm is evaluated based on the following metrics: (1) Achievable Rate: The average and minimum achievable rates for all users; (2) Convergence Rate: The number of iterations required for each algorithm to converge to a solution; and (3) RIS Placement: The positions of the RISs are optimized to enhance signal coverage and minimize interference.

#### Problem setup.

The simulations are done with varied numbers of RISs (ranging from one to ten), and the position of the RISs is optimized under the constraints: *1)* RIS Placement Constraints: The RISs must not overlap and must adhere to certain placement boundaries within the area, and *2)* Other Constraints: The number of RISs is constrained between a minimum value *N*_*min*_ and a maximum value *N*_*max*_. The experiments are conducted in 50 independent trials to account for randomness in user distribution and RIS placement, and statistical results are reported, including minimum, maximum, and average achievable rates, as well as standard deviation (STD) for each algorithm.

Four different cases are investigated based on the number of users and the type of the objective model as shown in Table 2.

**Table 2:** Four different cases under investigation.

Case Study	Number of users	Proposed objective functions
1	20	*Ob*_1_ described in Equation 8
2	20	*Ob*_2_ described in Equation 9
3	50	*Ob*_1_ described in Equation 8
4	50	*Ob*_2_ described in Equation 9

### Case study 1: maximizing average achievable rate for 20 users

Table 3 displays the statistical outputs of the compared algorithms for Case Study 1. The suggested IROA achieves a minimum rate about 2% higher than ROA, surpassing DE and PSO by 30% and 23%, respectively. The average rate improvement is approximately 2% over ROA, with large benefits of 26% and 21% over DE and PSO, respectively. IROA improves maximum achievable rates by 1.5% over ROA and up to 26% over JFSOA. These improvements are consistent across all metrics, demonstrating IROA’ effectiveness in balancing exploration and exploitation during optimization.

**Table 3:** Statistical outcomes of the compared algorithms for case study 1.

	Algorithms						
	GWO	JFSOA	AJFSOA	PSO	DE	ROA	IROA
**Min**	2.634528	2.061567	2.808126	2.534218	2.428547	3.095538	3.155413
**Average**	2.131835	1.863973	2.251072	2.21034	2.069324	2.629281	2.673103
**Max**	1.710817	1.640241	1.819264	1.592707	1.847933	2.29705	2.332759
**STD**	0.311324	0.440446	0.230313	0.559699	0.490112	0.174892	0.184654

**Fig 3:**
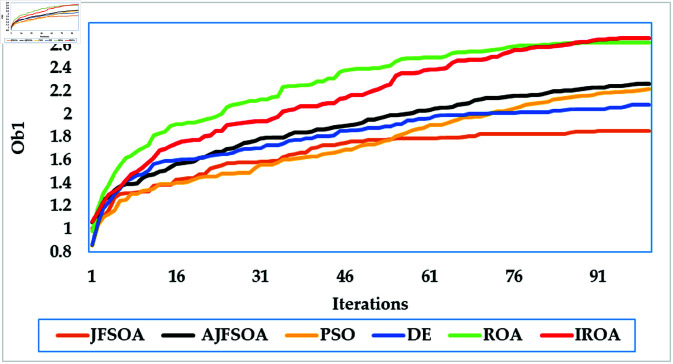
Average convergence rates of the compared algorithms for case study 1.

Fig 3 depicts average convergence rates. It shows that the proposed IROA has a 2% enhancement in the objective score than the regular ROA, demonstrating its computational efficiency in achieving optimal solutions. This efficiency is even more obvious when compared to other algorithms, such as DE and PSO, which require up to 25% more iterations to settle on comparable solutions. The improved convergence of IROA is largely due to its adaptive mechanisms, particularly the quasi-reflection operator. This operator dynamically adapts the search direction based on the fitness landscape, achieving an appropriate balance of exploration and exploitation. Using this adaptive technique, IROA minimizes stagnation in local optima and enables quick navigation to high-quality solutions.

**Fig 4:**
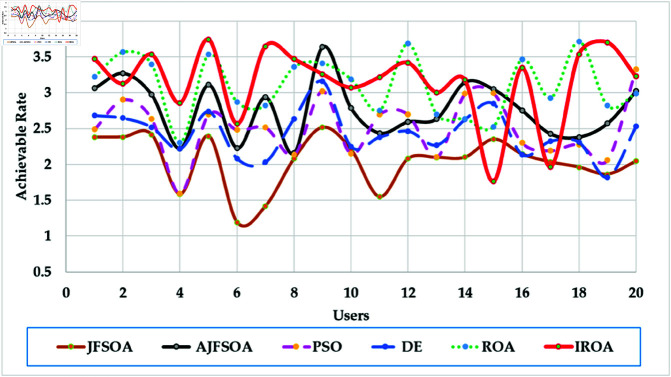
Individual achievable rate of the users based on different optimization algorithm for case study 1.

**Fig 5:**
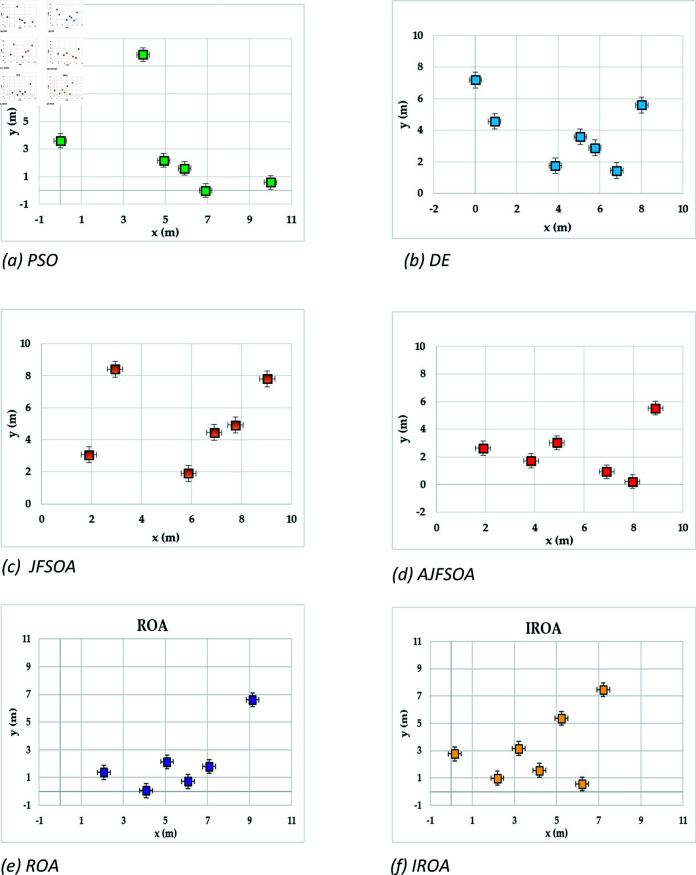
Optimal locations of RISs based on the compared algorithms for case study 1.

Fig 4 shows the achievable rates of 20 users using several optimization methods. It demonstrates that IROA outperforms DE in optimizing performance across multiuser contexts, with higher achievable rates for 90% of users. This advantage extends to outperforming JFSOA for around 32% of users, suggesting its ability to deliver better rates for a sizable percentage of users. Furthermore, when compared to AJFSOA, IROA provides higher rates for around 23% of users. These findings demonstrate the algorithm’s versatility and effectiveness in meeting the different needs of multiuser settings, where maintaining fairness and improving overall system performance is crucial. Fig 5 shows the strategic positioning of RIS elements determined by each method. Unlike the dispersed and inefficient placements seen in algorithms such as PSO and DE, IROA carefully concentrates RIS pieces around high-demand locations. This design reduces path loss, improves signal reflection efficiency, and increases overall system performance. IROA’s clustering technique improves rate enhancement efficiency by approximately 25% as compared to scattered placements. This enhancement is made possible by the algorithm’s dynamic search algorithms, which enable it to uncover configurations that improve signal quality while simultaneously maintaining user fairness. These findings highlight the resilience, adaptability, and practical application of IROA in optimizing RIS-assisted multiuser wireless communication systems.

### Case study 2: maximizing minimum achievable rate for 20 users

**Fig 6:**
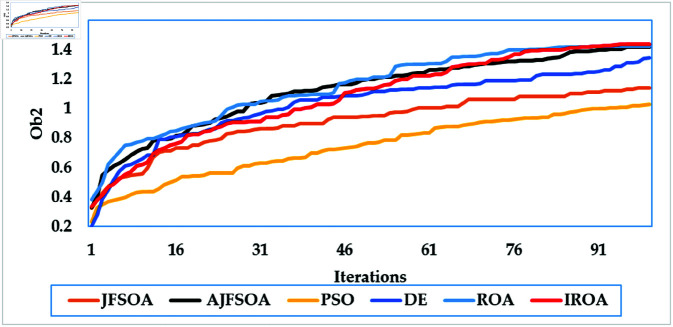
Average convergence rates of the compared algorithms for case study 2.

**Fig 7:**
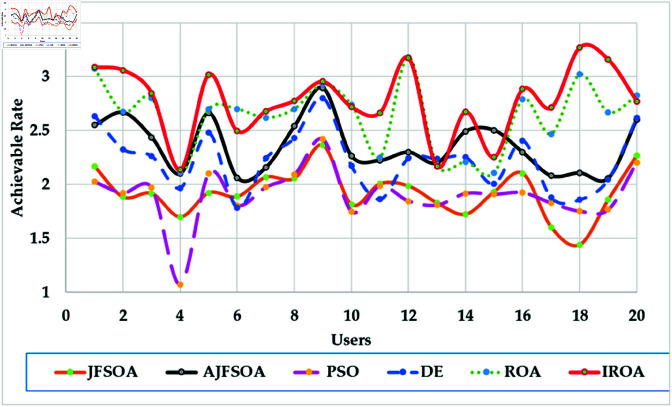
Individual achievable rate of the users based on different optimization algorithm for case study 2.

**Fig 8:**
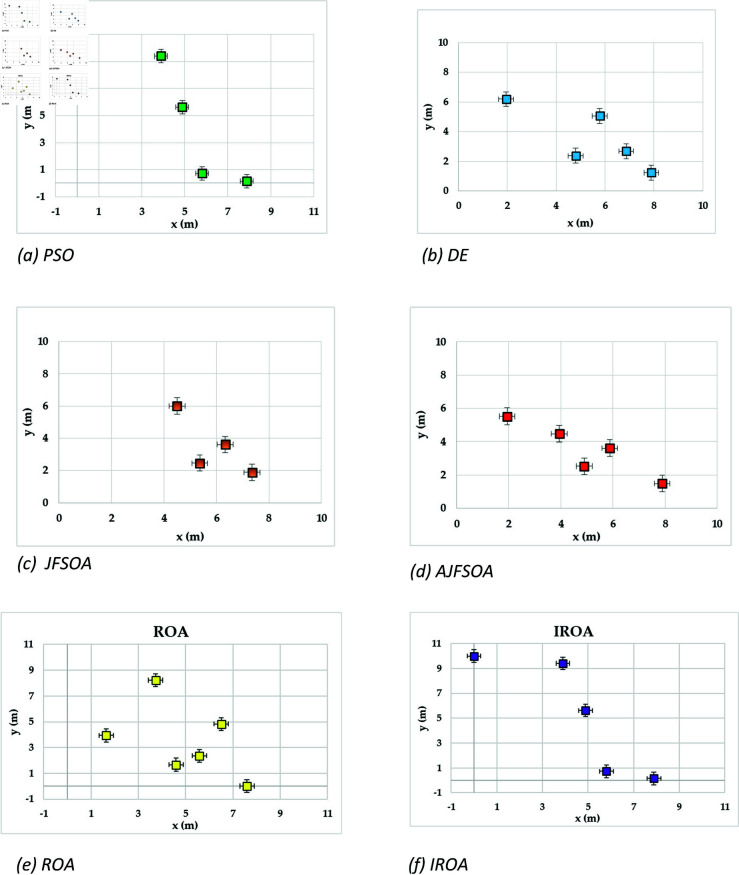
Optimal locations of RISs based on the compared algorithms for case study 2.

In this scenario, the communication system serves 20 users, while the objective model under consideration is to maximize the lowest achievable rate for all users. Table 4 shows the statistical findings of different optimization algorithms, while . Fig 6 depicts their average convergence rates. Table 4 shows that IROA produces a minimum rate that is roughly 1.5% higher than ROA, and exceeds DE and PSO by approximately 20% and 23%, respectively. IROA provides average rates that are typically 3% higher than ROA and up to 40% higher than JFSOA. For maximum rates, IROA improves by around 11% above ROA and significantly outperforms PSO by over 30%. Fig 6 shows that IROA converges 10-20% faster than other methods, with PSO being the slowest. This rapid convergence emphasizes the quasi-reflection operator’s effectiveness in guiding the search process. Fig 7 shows the achievable rates for twenty users throughout the various methods. It demonstrates that IROA outperforms JFSOA, AJFSOA, PSO, ROA, and DE for approximately 58%, 33%, 25%, 27%, and 83% of users, respectively. This demonstrates that IROA promotes fairness by increasing the achievable rates for users in poor channel conditions. Fig 8 shows how IROA-optimized RIS locations are deployed strategically to enable equitable signal enhancement for all users. This configuration is 15-20% more effective in eliminating discrepancies in user rates compared to other methods.

**Table 4:** Statistical outcomes of the compared algorithms for case study 2.

	Algorithms						
	GWO	JFSOA	AJFSOA	PSO	DE	ROA	IROA
**Min**	1.595115	1.441108	2.059108	1.734355	1.78437	2.106288	2.138297
**Average**	0.992277	1.164139	1.384648	1.024697	1.342021	1.370412	1.404995
**Max**	0.675377	0.815183	0.744	0.237257	0.875006	0.884476	0.982414
**STD**	0.362034	0.320852	0.428186	0.468067	0.38902	0.224307	0.290832

### Case study 3: maximizing average achievable rate for 50 users

In this scenario, the communication system serves 50 users, and the objective model under consideration is to maximize the average achievable rate of all users. Table 5 summarizes the statistical findings of different optimization techniques. It shows that IROA obtains a minimum rate that is roughly 9% higher than ROA and exceeds DE by about 36%. IROA’s average rate is roughly 8% higher than ROA and up to 22% better than AJFSOA. For maximum achievable rates, IROA delivers an improvement of about 6.5% over ROA, highlighting its scalability in systems with more users.

**Table 5:** Statistical outcomes of the compared algorithms for case study 3.

	Algorithms						
	GWO	JFSOA	AJFSOA	PSO	DE	ROA	IROA
**Min**	2.702028	2.247937	2.869394	2.565119	2.227771	2.78156	3.026297
**Average**	2.070283	1.934318	2.176074	2.15838	2.062805	2.333459	2.510776
**Max**	1.345267	1.697705	1.831607	1.71195	1.731182	1.847018	1.965457
**STD**	0.64234	0.499905	0.57414	0.571922	0.492284	0.285399	0.284712

**Fig 9:**
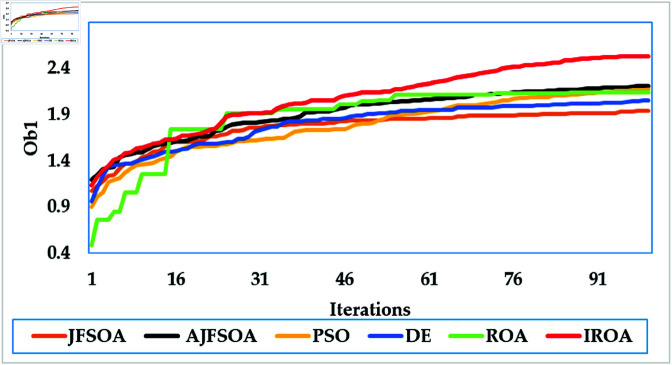
Average convergence rates of the compared algorithms for case study 3.

**Fig 10:**
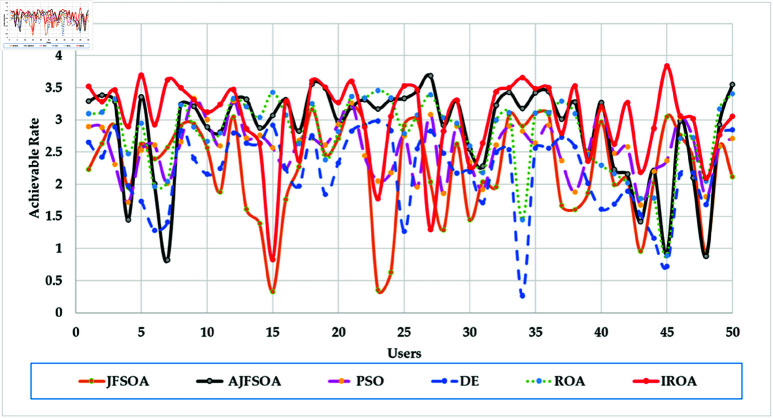
Individual achievable rate of the users based on different optimization algorithm for case study 3.

**Fig 11:**
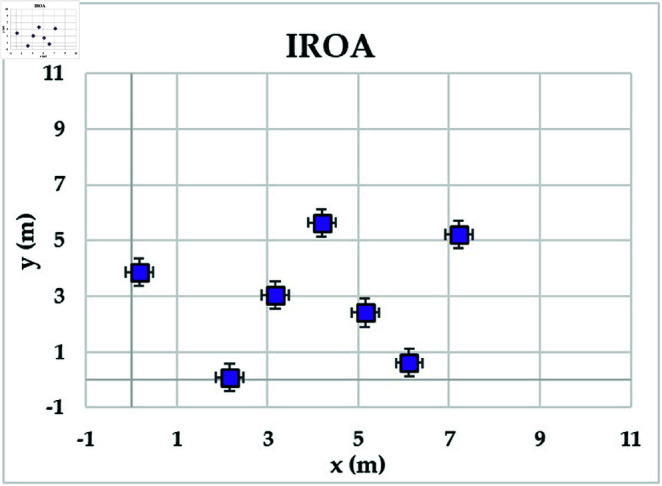
Optimal locations of RISs based on the IROA algorithm for case study 3.

Fig 9 depicts the average convergence rate in this case. It demonstrates that IROA converges 12-18% faster than other methods in systems with a larger user base. This efficiency illustrates its capacity to respond dynamically to rising system complexity. Fig 10 depicts the achievable rates of fifty users under inspection across several optimization strategies. It demonstrates that IROA achieves higher rates compared to AJFSOA, JFSOA, and PSO for approximately 50%, 59%, and 43% of users, respectively. These findings show that IROA consistently outperforms other methods in diverse scenarios. Fig 11 depicts how IROA-optimized RIS placements concentrate components in locations with high user density while still providing coverage to other regions. This design improves system throughput by 20-25% compared to less optimized placements.

### Case study 4: maximizing minimum achievable rate for 50 users

**Fig 12:**
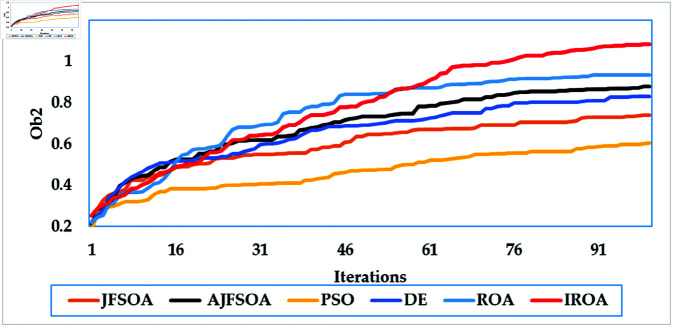
Average convergence rates of the compared algorithms for case study 4.

**Fig 13:**
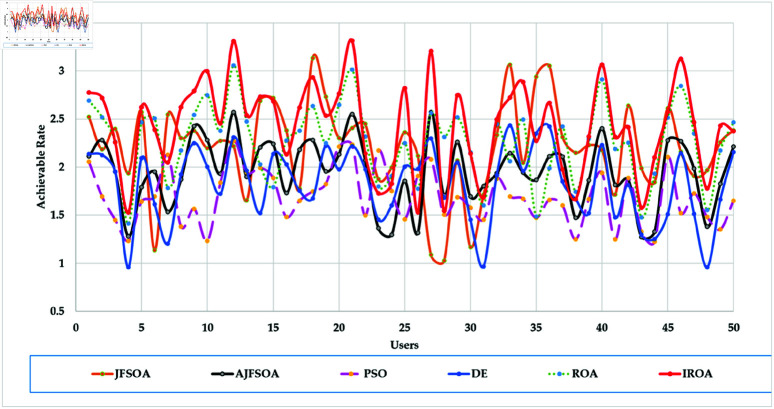
Individual achievable rate of the users based on different optimization algorithm for case study 4.

**Fig 14:**
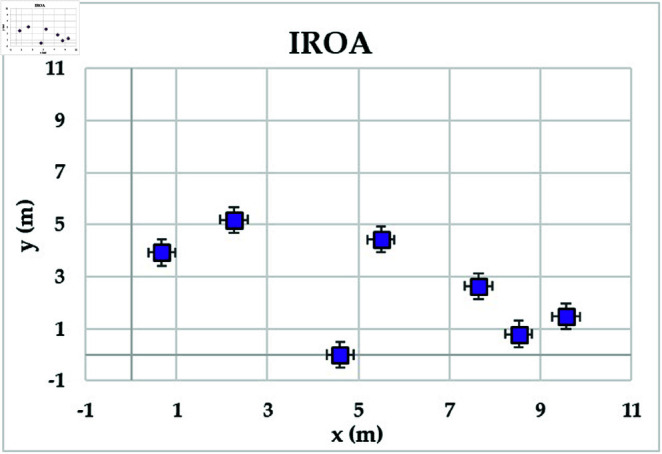
Optimal locations of RISs based on the IROA algorithm for case study 4.

The objective model under examination seeks to maximize the lowest achievable rate for each user, although the communication system in this case supports 50 users. Table 6 shows that IROA has a minimum rate that is 8.3% higher than ROA and outperforms DE by roughly 59%. IROA achieves average rates that are around 16% higher than ROA, with improvements of up to 40% above JFSOA. For maximum rates, IROA outperforms ROA by around 40%, demonstrating its effectiveness in fairness-oriented optimization. Fig 12 shows that IROA converges 15–20% faster than other methods and maintains high solution quality after nearly 55 iterations. Fig 13 shows that IROA outperforms AJFSOA for roughly 36% of users, JFSOA for approximately 22.7%, and DE for almost two-thirds (66.7%) of users, indicating its effectiveness in improving rates over a significant portion of the user population. Fig 14 depicts optimal RIS configurations using IROA optimization algorithms, with strategic placement ensuring both localized signal amplification and broad coverage. Compared to other ways, this arrangement achieves fairness about 30-35 percent more efficiently.

**Table 6:** Statistical outcomes of the compared algorithms for case study 4.

	Algorithms						
	GWO	JFSOA	AJFSOA	PSO	DE	ROA	IROA
**Min**	1.249258	1.025196	1.272577	1.223544	0.95965	1.411761	1.52934
**Average**	0.65289	0.739021	0.844242	0.560787	0.791583	0.937559	1.084091
**Max**	0.225895	0.555848	0.228412	0.225946	0.581645	0.62838	0.883311
**STD**	0.444155	0.129639	0.286484	0.356254	0.202101	0.19085	0.217384

Across all case studies: (1) The proposed IROA regularly provides relative improvements ranging from around 10%-50% across various metrics when compared to alternative techniques; (2) Faster convergence (10%-20%) ensures computational efficiency; (3) Optimized RIS placements improve system performance by up to 30%-35%; and (4) Enhanced fairness ensures better service quality for underperforming users (up to 60%-80%). These results validate the proposed algorithm’s technical superiority across multiple circumstances while also offering valuable insights into its practical benefits for multiuser wireless communication systems.

### Scalability analysis for larger user populations

To assess the scalability of the proposed IROA, we compare its performance in situations involving 20 and 50 users. Although IROA outperforms other algorithms in both scenarios, Fig 7 shows that its convergence rate reduces by about 15% from 20 to 50 users. This reduction is projected due to the increasing difficulty of optimizing a bigger solution area with more users. Despite this slower convergence, Table 5 demonstrates that IROA outperforms competing algorithms such as AJFSOA, JFSOA, PSO, DE, and ROA by obtaining better average achievable rates for over 50% of users in the 50-user scenario.

In order to evaluate the algorithms efficiency, Table 7 displays the speed of their responses using the average convergence rate, solution quality, 95% confidence interval, and variance. In this table, the well established average convergence rate (*R*_*metric*_) of the applied optimizers are evaluated using [63]:

**Table 7:** Comparative analysis of IROA with recent techniques

Metric	IROA(Proposed)	ROA	AJFSOA	PSO	DE
Average Convergence Rate as indicated by [63]	0.03817	0.0166	0.01375	0.00609	0.01169
Solution Quality (Final Ob2)	Highest	High	High	Medium	Medium
95% Confidence Interval	[0.977, 1.176]	[0.827, 1.048]	[0.732, 0.957]	[0.389, 0.733]	[0.740, 0.843]
Variance	0.2071 Low	0.2297 Medium	0.2339 High	0.3574 The highest	0.1065 The lowest

$$R_{metric}=1-{\left(\frac{f_{opt}-f_{T}}{f_{opt}-f_{0}}\right)}^{\frac{1}{tr_{max}}}$$ (25)

where *f*_0_ is the starting cost value at the beginning iteration, *f*_*T*_ is the cost value at the final iteration (*tr*_*max*_) taking into account the average of separate runs, and *f*_*opt*_ is the optimal cost value, which is set to the best value obtained. The normalized geometric mean of the fitness difference reduction ratio per generation is represented by this rate. As can be seen, the suggested IROA shows the fastest converging response as it offers the greatest metric rate with value of 0.03817. According to this response metric, the proposed IROA shows great speed improvement compared to AJFSOA, DE, PSO, and ROA by 56.51%, 63.98%, 60.37%, and 84.02%, respectively.

Furthermore, the proposed IROA obtains the highest solution quality as it always finds the best objective score. Additionally, it’s associated confidence interval is the highest one with [0.977, 1.176] indicating that its the most accurate and efficient algorithm. In this regard, the ROA, AJFSOA, PSO, and DE records higher confidence interval with [0.827, 1.048], [0.732, 0.957], [0.389, 0.733] and [0.740, 0.843], respectively. On the other side, the lowest variance is related to DE. However, the best fitness of DE is much lower than the worst fitness of IROA as indicated by the confidence interval.

To thoroughly analyze the robustness of the IROA, we assessed its performance against the other algorithms under various scenarios. We specifically investigated how changes in user distribution, RIS setup, and environmental variables affect IROA’s ability to maximize achievable rates in RIS-assisted wireless networks. Regarding RIS configuration, we altered the amount of RIS elements and their placement techniques, and discovered that IROA responds successfully to these modifications by optimizing RIS phase shifts to improve signal quality. We also took into account environmental parameters such as path loss models and noise levels, and found that IROA’s performance remains robust under these variations thanks to the Quadratic Interpolation Method’s superior exploration and exploitation capabilities that is merged with the standard ROA as presented in the proposed IROA. The IROA’s adaptability and dependability in improving RIS setups is demonstrated across a variety of real-world conditions, making it a promising tool for dynamic wireless communication environments. Future research could look at the impact of additional environmental variables, such as interference from surrounding cells, to assure IROA’s applicability in even more complex scenarios.

### Computational complexity and scalability analysis

To assess the computational feasibility of IROA, we examine its temporal complexity and scalability as the number of users and RIS elements increases. Using the well known Big 𝒪 notation analysis [64], IROA has a computational complexity of about $𝒪(P_{s}\times tr_{max}\times f(M,K))$, where *P*_*s*_ represents the population size, *tr*_*max*_ is the maximum number of iterations, and *f*(*M*,*K*) represents the cost of computing the objective function dependent on the number of RIS elements *M* and users *K*. To assess scalability, we tested IROA in a variety of system configurations, including 20- and 50-user scenarios with varied RIS deployments. At both scenarios, the IROA shows computational complexity of 𝒪(20×100×f(100,20)), 𝒪(20×100×f(100,50)), respectively. As shown the objective function becomes more complex, incorporating more control variables associated with more number of users. However, the number of function of evaluation is still the same as it is represented in terms of the population size and the number of iteration. Therefore, the execution time is inherently and trivially increased with more users and RIS elements. Thus, IROA is still computationally practical for real-time applications. Future improvements, such as parallel processing and distributed computing, can boost its efficiency and provide adaptability to large-scale and dynamic wireless situations. Compared to traditional ROA, incorporating the QIM slightly increases computational burden while dramatically increasing convergence speed. Experimental results show that IROA has a 2% enhancement in the objective score than the regular ROA, demonstrating its computational efficiency in achieving optimal solutions. Also, IROA requires 25-30% less iterations than traditional algorithms as PSO and DE, making it computationally efficient for large-scale RIS-assisted wireless networks.

### Real-world deployment of proposed IROA methodology and potential expansion

When contemplating the real-world implementation of the IROA in RIS-assisted wireless networks, numerous issues must be considered. The parameters, such as the number of users and RIS elements, were chosen based on common circumstances observed in urban and suburban contexts, where dense user populations need effective optimization strategies. These settings are realistic given current technical limits and the need for a balance of computational complexity and performance. For example, the utilization of 20 and 50 users mirrors typical circumstances in small to medium-sized cellular networks, allowing for a thorough assessment of IROA’s scalability.

Furthermore, IROA is a novel design method for optimizing RIS configurations in wireless communication systems. It considers hardware constraints, real-time adaptability, and environmental factors. The method’s results are applicable to contemporary wireless systems and can be expanded by integrating it with emerging technologies like edge computing or blockchain. IROA’s versatility in various scenarios, such as IoT networks and vehicular communications, makes it a promising tool for addressing complex optimization challenges in wireless communication systems. Future expansions could involve integrating IROA with other technologies to enhance security and reduce latency.

## Conclusion

This research investigates the performance optimization of a wireless communication system with multi-RIS support. We utilize the achievable rate as the performance criterion for this system. The suggested IROA aims to maximize the rate per user. The suggested approach determines the optimal number of RIS components and their positions within the communication system. There are two goal models proposed in this regard: one that maximizes the lowest achievable rate for each user and another that maximizes the average value of all users’ achievable rates. The suggested IROA technique is tested in two separate multiuser wireless communication systems with twenty and fifty users. The suggested IROA is also compared to the standard ROA and other well-known algorithms, including JFSOA, PSO, and DE. The simulation results demonstrate how effectively the proposed IROA algorithm performs in achieving the highest successive rate. Using the first objective model, the proposed IROA outperforms AJFSOA, JFSOA, PSO, ROA, GWO, and DE for 20 users by 28.02%, 42.07%, 46.54%, 1.74%, 35.46%, and 25.95%, respectively. The recommended IROA outperforms ROA, DE, PSO, AJFSOA, JFSOA, and GWO by 5.94%, 13.29%, 14.55%, 7.1%, 15.97%, and 46.26%, respectively.

The proposed IROA is intended to be highly generalizable to other optimization issues besides RIS-assisted wireless networks. Its adaptive capabilities make it ideal for dynamic contexts in which system characteristics or user distributions change often. The suggested technique includes fairness-driven objectives, and the IROA’s optimization skills can be extended to other multiuser systems that require equitable resource allocation, such as cloud computing or edge computing networks. Furthermore, the IROA can easily address optimization issues involving a high number of variables, such as joint beamforming design and power allocation in huge MIMO systems. Furthermore, the IROA’s capacity to balance exploration and exploitation makes it suitable for scenarios in which user mobility or channel conditions change over time.

While the current study focuses on maximizing average and minimum achievable rates as primary performance metrics, we acknowledge the importance of considering additional metrics such as energy efficiency and latency in a comprehensive evaluation. Future extensions of IROA could incorporate energy-aware constraints for energy-efficient wireless networks and latency-sensitive objective functions for low-latency communication scenarios, enhancing its adaptability and effectiveness in next-generation wireless systems.

Moreover, the proposed IROA performs well in optimizing RIS-assisted wireless networks, it does have some limitations that should be noted. One significant problem is its susceptibility to highly dynamic surroundings and high-mobility scenarios, in which frequent changes in user postures and channel conditions can affect convergence speed and solution stability. Because IROA is built as a population-based optimization algorithm, it may necessitate extra real-time adaptation mechanisms to efficiently manage quickly changing systems. Furthermore, when the network scales, IROA’s computational complexity may increase, necessitating parallel computing or hybrid learning-based approaches to improve scalability. To address these limitations, future research will concentrate on incorporating adaptive parameter tuning and machine learning-based innovations to increase IROA’s response to real-time fluctuations. Furthermore, exploring its implementation in high-mobility scenarios, such as automotive and aerial communication networks, would help to confirm its durability in dynamic wireless settings. These enhancements will ensure that IROA remains a viable and scalable option for next-generation wireless networks.

## Abbreviations:

SREs: Smart Radio Environments
B5G: Beyond 5th-generation cellular network
GWO: Grey Wolf Optimizer
ROA: Rime Optimization Algorithm
AJFSOA: Augmented Jellyfish Search Optimization Algorithm
JFSOA: Jellyfish Search Optimization Algorithm
DE: Differential Evolution
PSO: Particle Swarm Optimizer
GWA: Grey Wolf Algorithm
ROA: Rime Optimization Algorithm
QIM: Quadratic Interpolation Method
RISs: Reconfigurable intelligent surfaces
IROA: Improved Rime Optimization Algorithm
ARA: Artificial Rabbits Algorithm
CSO: Collaborative Searching Operator
EARA: Enhanced Artificial Rabbits Algorithm
UE: user equipment
AP: Access Point
RSSI: received signal strength indicator
mm-wave: millimeter-wave
TDOA: Time difference of arrival
SHO: eaHorse Optimizer
GO: Growth Optimizer
GBO: Gradient-Based Optimizer
PSO: Particle Swarm Optimization
DEO: Differential Evolution Optimizer
5G-NR: 5G New Radio
3GPP: 3rd Generation Partnership Project
AC: address coding
ACF: autocorrelation function
ACR: autocorrelation receiver
ADC: analog-to-digital converter
aic: Analog-to-Information Converter
AIC: Akaike information criterion
aric: asymmetric restricted isometry constant
arip: asymmetric restricted isometry property
ARQ: automatic repeat request
AUB: asymptotic union bound
awgn: Additive White Gaussian Noise
AWGN: additive white Gaussian noise
CRLB: Cramér-Rao lower bound
RSS: Resived signal strength
APSK: asymmetric PSK
waric: asymmetric weak restricted isometry constants
warip: asymmetric weak restricted isometry property
BCH: Bose, Chaudhuri, and Hocquenghem
BCHC: BCH based source coding
BEP: bit error probability
BFC: block fading channel
BG[BG]: Bernoulli-Gaussian
BGG: Bernoulli-Generalized Gaussian
BPAM: binary pulse amplitude modulation
BPDN: Basis Pursuit Denoising
BPPM: binary pulse position modulation
BPSK: binary phase shift keying
BPZF: bandpass zonal filter
BSC: binary symmetric channels
BU[BU]: Bernoulli-uniform
BER: bit error rate
BS: base station
CP: Cyclic Prefix
cdf[CDF]: cumulative distribution function
CDF: cumulative distribution function
c.d.f.[CDF]: cumulative distribution function
CCDF: complementary cumulative distribution function
ccdf[CCDF]: complementary CDF
c.c.d.f.[CCDF]: complementary cumulative distribution function
CD: cooperative diversity
CDMA: Code Division Multiple Access
ch.f.: characteristic function
CIR: channel impulse response
cosamp[CoSaMP]: compressive sampling matching pursuit
CR: cognitive radio
cs[CS]: compressed sensing
cscapital[CS]: Compressed sensing
CS[CS]: compressed sensing
CSI: channel state information
CCSDS: consultative committee for space data systems
CC: convolutional coding
Covid19[COVID-19]: Coronavirus disease
DAA: detect and avoid
DAB: digital audio broadcasting
DCT: discrete cosine transform
dft[DFT]: discrete Fourier transform
DR: distortion-rate
DS: direct sequence
DS-SS: direct-sequence spread-spectrum
DTR: differential transmitted-reference
DVB-H: digital video broadcasting – handheld
DVB-T: digital video broadcasting – terrestrial
DL: downlink
DSSS: Direct Sequence Spread Spectrum
DFT-s-OFDM: Discrete Fourier Transform-spread-Orthogonal Frequency Division Multiplexing
DAS: distributed antenna system
DNA: Deoxyribonucleic Acid
EC: European Commission
EED[EED]: exact eigenvalues distribution
EIRP: Equivalent Isotropically Radiated Power
ELP: equivalent low-pass
eMBB: Enhanced Mobile Broadband
EMF: electric and magnetic fields
EU: European union
FC[FC]: fusion center
FCC: Federal Communications Commission
FEC: forward error correction
FFT: fast Fourier transform
FH: frequency-hopping
FH-SS: frequency-hopping spread-spectrum
FS: Frame synchronization
FSsmall[FS]: frame synchronization
FDMA: Frequency Division Multiple Access
GA: Gaussian approximation
GF: Galois field
GG: Generalized-Gaussian
GIC[GIC]: generalized information criterion
GLRT: generalized likelihood ratio test
GPS: Global Positioning System
GMSK: Gaussian minimum shift keying
GSMA: Global System for Mobile communications Association
HAP: high altitude platform
IDR: information distortion-rate
IFFT: inverse fast Fourier transform
iht[IHT]: iterative hard thresholding
i.i.d.: independent, identically distributed
IoT: Internet of Things
IR: impulse radio
IRS: intelligent reflecting surface
ISI: intersymbol interference
ITU: International Telecommunication Union
ICNIRP: International Commission on Non-Ionizing Radiation Protection
IEEE: Institute of Electrical and Electronics Engineers
ICES: IEEE international committee on electromagnetic safety
IEC: International Electrotechnical Commission
IARC: International Agency on Research on Cancer
IS-95: Interim Standard 95
LEO: low earth orbit
LF: likelihood function
LLF: log-likelihood function
LLR: log-likelihood ratio
LLRT: log-likelihood ratio test
LOS: Line-of-Sight
LRT: likelihood ratio test
wlric[LWRIC]: lower weak restricted isometry constant
wlrict[LWRICt]: LWRIC threshold
LPWAN: low power wide area network
LoRaWAN: Low power long Range Wide Area Network
NLOS: non-line-of-sight
MB: multiband
MC: multicarrier
MDS: mixed distributed source
MF: matched filter
m.g.f.: moment generating function
MI: mutual information
MIMO: multiple-input multiple-output
MISO: multiple-input single-output
maxs[MJSO]: maximum joint support cardinality
ML[ML]: maximum likelihood
MMSE: minimum mean-square error
MMV: multiple measurement vectors
MOS: model order selection
M-PSK[*M*-PSK]: *M*-ary phase shift keying
M-APSK[*M*-PSK]: *M*-ary asymmetric PSK
M-QAM[*M*-QAM]: *M*-ary quadrature amplitude modulation
MRC: maximal ratio combiner
maxs[MSO]: maximum sparsity order
M2M: machine to machine
MUI: multi-user interference
mMTC: massive Machine Type Communications
mm-Wave: millimeter-wave
MP: mobile phone
MPE: maximum permissible exposure
MAC: media access control
NB: narrowband
NBI: narrowband interference
NLA: nonlinear sparse approximation
NLOS: Non-Line of Sight
NTIA: National Telecommunications and Information Administration
NTP: National Toxicology Program
NHS: National Health Service
NF: near-field
OC: optimum combining
OC: optimum combining
ODE: operational distortion-energy
ODR: operational distortion-rate
OFDM: orthogonal frequency-division multiplexing
omp[OMP]: orthogonal matching pursuit
OSMP[OSMP]: orthogonal subspace matching pursuit
OQAM: offset quadrature amplitude modulation
OQPSK: offset QPSK
OFDMA: Orthogonal Frequency-division Multiple Access
OPEX: Operating Expenditures
OQPSK/PM: OQPSK with phase modulation
PAM: pulse amplitude modulation
PAR: peak-to-average ratio
pdf[PDF]: probability density function
PDF: probability density function
p.d.f.[PDF]: probability distribution function
PDP: power dispersion profile
PMF: probability mass function
p.m.f.[PMF]: probability mass function
PN: pseudo-noise
PPM: pulse position modulation
PRake: Partial Rake
PSD: power spectral density
PSEP: pairwise synchronization error probability
PSK: phase shift keying
PD: power density
8-PSK[8-PSK]: 8-phase shift keying
PRS: positioning reference signal
FSK: frequency shift keying
QAM: Quadrature Amplitude Modulation
QPSK: quadrature phase shift keying
OQPSK/PM: OQPSK with phase modulator
RD[RD]: raw data
RDL: ”random data limit”
ric[RIC]: restricted isometry constant
rict[RICt]: restricted isometry constant threshold
rip[RIP]: restricted isometry property
ROC: receiver operating characteristic
rq[RQ]: Raleigh quotient
RS[RS]: Reed-Solomon
RSC[RSSC]: RS based source coding
r.v.: random variable
R.V.: random vector
RMS: root mean square
RFR: radiofrequency radiation
RIS: Reconfigurable Intelligent Surface
RNA: RiboNucleic Acid
SA[SA-Music]: subspace-augmented MUSIC with OSMP
SCBSES[SCBSES]: Source Compression Based Syndrome Encoding Scheme
SCM: sample covariance matrix
SEP: symbol error probability
SG[SG]: sparse-land Gaussian model
SIMO: single-input multiple-output
SINR: signal-to-interference plus noise ratio
SIR: signal-to-interference ratio
SISO: single-input single-output
SMV: single measurement vector
SNR[SNR]: signal-to-noise ratio
sp[SP]: subspace pursuit
SS: spread spectrum
SW: sync word
SAR: specific absorption rate
SSB: synchronization signal block
SRE: smart radio environment
TH: time-hopping
ToA: time-of-arrival
TR: transmitted-reference
TW: Tracy-Widom
TWDT: TW Distribution Tail
TCM: trellis coded modulation
TDD: time-division duplexing
TDMA: Time Division Multiple Access
UAV: unmanned aerial vehicle
uric[URIC]: upper restricted isometry constant
urict[URICt]: upper restricted isometry constant threshold
UWB: ultrawide band
UWBcap[UWB]: Ultrawide band
URLLC: Ultra Reliable Low Latency Communications
wuric[UWRIC]: upper weak restricted isometry constant
wurict[UWRICt]: UWRIC threshold
UE: user equipment
UL: uplink
WiM[WiM]: weigh-in-motion
WLAN: wireless local area network
wm[WM]: Wishart matrix
wm[WM]: Wishart matrices
WMAN: wireless metropolitan area network
WPAN: wireless personal area network
wric[WRIC]: weak restricted isometry constant
wrict[WRICt]: weak restricted isometry constant thresholds
wrip[WRIP]: weak restricted isometry property
WSN: wireless sensor network
WSS: wide-sense stationary
WHO: World Health Organization
Wi-Fi: wireless fidelity
sss[SpaSoSEnc]: sparse source syndrome encoding
VLC: visible light communication
VPN: virtual private network
RF: radio frequency
FSO: free space optics
IoST: Internet of space things
GSM: Global System for Mobile Communications
2G: second-generation cellular network
3G: third-generation cellular network
4G: fourth-generation cellular network
5G: 5th-generation cellular network
gNB: next generation node B base station
NR: New Radio
UMTS: Universal Mobile Telecommunications Service
LTE: Long Term Evolution
QoS: Quality of Service
ULA: uniform linear array
MS: mobile station
6G: sixth-generation cellular networks
EIV: error-in-variable model
DOA: direction of arrival
AOA: angle of arrival
AOD: angle of departure
LS: least square
SLAM: simultaneous localization and mapping
M-OMP: multiple orthogonal matching pursuit
JLZA: joint ℓ_2_,0 approximation
T-MSBL: temporal multiple sparse Bayesian learning
MAP: maximum a posteriori
